# Instructions to Mothers and Nurses on the Management of Children in Health and Disease

**Published:** 1825-10-01

**Authors:** 


*Instructions to Mothers and Nurses, on the Management of Children in Health and Disease; comprehending Directions for regulating their Diet, Dress, Exercise, and Medicine; with a Variety of Prescriptions adapted to the Use of the Nursery, and an Index of Medical Terms.* By James Kennedy, M. D. Small octavo, pp. 329. Glasgow and London, 1825.

When it is recollected, that one half of those who enter into the busied scene of human existence, depart from it before the completion of their eighth year, and that, of a thousand infants born in London, six hundred and fifty die previous to the age of ten, it will be admitted that the subject which Dr. Kennedy has chosen possesses considerable interest, and requires a happy combination of talent observation, and judgment. Such a work was much wanted. The management of children, from birth to puberty, is a most important consideration, and especially so on these particular subjects, where prejudice and ignorance, anxiety, and affection, are in full operation. Correct opinions, relative to diet, dress, suckling, dentition, air, exercise, nursing, medicine, and other matters equally momentous, can only be gained by long experience; and, until such experience be obtained, a youthful practitioner must either remain comparatively ignorant of what he ought to know, or have recourse to books to enlighten his mind, which may be not unaptly termed a *royal road* to sound practical knowledge. Such a *royal road*, Dr. Kennedy has opened, by the publication of his valuable instructions, which are replete with varied and extensive information, scientific and judicious views, and acute and even philosophical observations. The title-page does the author injustice; it seems to imply, he has only written, in a popular manner, to suit the meridian of mothers and nurses; but it is directly the reverse, as we shall attempt to prove: he treats every subject with precision, science, and judgment, and he that can read his pages, and deny that he is an able physician and instructive author, must be either destitute of candour, or defective in understanding.

Before we proceed farther, it is but just to allow Dr. Kennedy to explain what objects he had in view, when he took up his pen.

"The doctrines inculcated in this volume have been deduced from attentive observation of the effects of management and of medicine on the manifold circumstances of children in health and disease:—they are the results of extensive reading, of considerable practical experience, and of much reflection; and, though communicated in a didactic style, are submitted with deference to the judgment of parents, and with the sincerest solicitude that their applications may be found beneficial.

"Agreeably to its natural arrangement, the work is divided into two parts:—the first, after an introductory description of the three first epochs of life, includes a concise illustration of the modes whereby the chief diversities of food, dress, air, and exercise, can be so managed by parents, as to promote the comfort, strengthen the constitution, and remove or mitigate the various ailments of children: in the second, are enumerated the methods of treating their disease to a definite extent, beyond which any interference of the unskilful might prove injurious or fatal: more than this would tend only to perplex, or lead into dangerous security, those to whom the guardianship of young persons may be intrusted." *Preface*, vii.

We cannot pretend to analyse a work of this nature, but must content ourselves with the selection of such parts, as will convey useful information. Full justice is done to the fœtal state, from its primitive germ to complete evolution, and premising, once for all, that the physiology of every subject is extensively entered into, with the introduction of much useful collateral matter, it is our object to pass these over, and only to bring forward what is applicable to real practical utility. From the second, or medical portion, no extracts will be needed; for, although cautious and judicious, it is intended for mothers and nurses, and necessarily ceases at that period of diseases, when the services of professional men are wanted. It may be urged, that mothers and nurses should never presume to tamper with the incipient ailments or complaints of children; to a certain extent, this is true,—but, since they will do so, surely it is desirable to furnish them with safe and beneficial instructions: and this is evidently the design of the author, who fails not to enjoin an early recourse to practitioners, in all cases apparently serious. Human life is too valuable to be trifled with; the inflammatory diseases of children advance with fearful rapidity; the loss of a day or two is pregnant with danger; and if this work teach females instantly to comprehend their nature, and to employ active means, it cannot but render an essential service to nurseries and parlors. The treatment of the acute affections of these engaging beings, demands attentive consideration and nice discrimination, and every natural and incidental circumstance, peculiar to them, is to be duly estimated. A medical man, while commenting on the illness of a child, may lay considerable stress on its rapid respiration and accelerated pulse, and to this there is no objection, provided he remember that, in the first year of life, the child breathes 35 times in a minute; in the second year, 25 times; at puberty, only 20 times; and that, in a new-born infant, placidly sleeping, the pulse is 140 in a minute, in the first year; 124, in the second; 110 and 96 in the third and fourth years; but, if these influential facts be unknown or forgotten, actual health may be mistaken for serious indisposition. This is merely stated for the sake of general illustration.

The following valuable case, cited by Dr. Kennedy, deserves all the publicity which our Journal can afford; we attach much importance to it, as indicative of the influence of local and foreign irritation, and as demonstrative of the occasional dangers resulting from the transplanting of teeth. This case was published many years ago, by Dr. Watson, in the Medical Transactions, but that work is now comparatively scarce; and we are persuaded, that the re-publication of the case, at this period, will impart advantage to many, who either never heard of it, or know not where it is to be found. The recordation of facts, is the imperious duty of every man: they correct the pride of opinion, and are the only sure land-marks against error.

"An unmarried lady, twenty-one years of age, had one of the incisive teeth in her upper jaw affected with the carious decay, arising from an unknown cause: it was extracted, and its place very dexterously supplied by a like tooth from another young woman, who, upon a most rigid examination for the purpose, appeared to be in excellent health. The implanted tooth very rapidly took a firm hold, and soon bid fair to be of great service and ornament. In about a month, however, the mouth became painful, the gums inflamed, discoloured, and ulcerated. The ulceration spread very fast, the gums of the upper jaw were destroyed, and the sockets left bare. Before the end of another month, the ulceration stretched outwardly over the upper lip and nose, and inwardly to the cheeks and throat, which were corroded by large, deep, and fetid sores. The gums soon became carious, several of the teeth successively dropped out, and these at length were followed by the implanted tooth, which had hitherto remained firm in its place. About this time also, blotches appeared in the face, neck, and various parts of the body, many of which became painful and extensive ulcers:—a considerable degree of fever, apparently hectic, was excited:—a copious and fetid discharge flowed from the mouth and throat, and impeded sleep:—and the soreness of the parts which perform deglutition, prevented a sufficiency of nourishment from being swallowed.—Medicines, exhibited in every possible form that science matured by experience could suggest, failed altogether of removing or even mitigating the unhappy sufferer's distresses:—the virulent taint or putrescent tendency established in the system, though occasionally driven back, as often rallied, and ultimately prevailed:—the patient fell a victim to it, in the greatest anguish and misery.—The person from whom the tooth had been taken, had all along continued to enjoy the most perfect health; she was frequently and scrutinously examined, without a single trace of disease being discovered either in her person or constitution." 61—62.

It is the duty of every mother to suckle her infant, whenever she is able; and if we knew a mother who was averse from such a delightful office, we would address her in the irresistible and energetic language of an ancient Greek philosopher:—"Woman! did you support this child with your blood, before you saw it, or knew it would live; and do you now refuse, when warm with life, and blooming with beauty, it smiles upon you, looks you in the face, expands its little hands, and seems to beg you will not forsake it?"

"Infants should be put to the breast in four or five hours after their birth:—in the interval, they may get some sweetened water or other very mild thin fluid, for the purpose of removing whatever viscid matter may be adhering to the internal surfaces of the gullet. If twenty-four or more hours shall elapse before the babe is permitted to suck the mother, her breasts become distended with her milk, the act of sucking occasions her acute pain, and the exertions of the infant to imbibe its new beverage often prove the cause of painful chaps in the nipple. Simple as this circumstance of being early sucked, may appear to be, it contributes essentially to making the milk-fever milder, and sometimes altogether prevents its occurrence. Such delay, too, excludes the young one from obtaining the full advantages which nature has bountifully provided for it, in the use of this first milk. Even the parent's own health and the activity of her lactiferous functions depend, in no small measure, on her breasts being excited by an early act of sucking. This has the beneficial effect, also, of conducing not remotely to the milk's increase and the improvement of its quality,—both which indeed, especially its quantity, are generally commensurate with the degree of vitality possessed by its secreting organs :—besides, during the time it remains in the breasts, it undergoes an elaboration by which its nutritive virtues are augmented and matured."

"Preparatively to putting her young one to suck for the first time,—which may be in three or four hours after its birth,—the mother should have her breasts carefully fomented,—primarily with tepid water and soap of the blandest kind,—and then with a lotion composed of milk and water in equal proportions, slightly sweetened, and warmer by a few degrees than the temperature of her own person. Such means, notwithstanding their simplicity, contribute much to accelerate the secretion of milk, make its progress less distressing, and, by removing the bitter exudation naturally deposited on the nipple, facilitate the suckling's first exertions at obtaining the vital fluid destined for the nourishment of its helpless days. While this is being done, she should retain a lying posture in the bed, and be exposed to the least possible annoyance, or fatigue, or danger of cold. Without changing her attitude, the babe may now be admitted to her breast, and will, in general, begin attempting to suck. If, however, it be listless and reject the nipple, it ought to be withdrawn, so as not to disquiet or exhaust the parent with its refusals or unavailing efforts : but, in a short time, a new trial of its inclinations may be made. When mothers cannot give the breast, except in a sitting posture, they should be raised with the gentlest caution, have themselves comfortably supported with pillows, and secured by proper coverings from sustaining injury by the influences of external air.

"Infants, at the beginning, are able to obtain but little milk :—this little, however, is of the highest use for promoting objects in them, which have already been explained. In each successive endeavour, from its exciting the nerves and vessels of the breast, the supply becomes more copious, and they imbibe it in greater abundance and with the greatest ease :—in the end, it constitutes a most delicious fare on which they feast with advantage and delight." 82—86.

The apparel of a child should be without complication :—it ought to be light, soft, loose, warm, and most simple, and never extend more than two or three inches beyond the feet. The use of pins should be superseded by fine tapes, which are always safe, and equally convenient. Dr. Underwood, distinguished for his professional experience, has sufficiently reprobated the employment of pins, by the following melancholy narration. "A gentlewoman, many years ago informed me," he says, "that one of her children, after long and incessant crying, fell into strong convulsions, which her physician was at a loss to account for; nor was the cause discovered till after death, when, on the cap being taken off, which had not been changed on account of the child's illness, a small pin was discovered, sticking up to the head, in the large fontanelle."

The use of cradles for children appears to have been common in the earliest ages, and was probably derived from the practice of barbarians, who placed their offspring in hurdles, which were attached to the branches of trees. Plautus, who wrote two thousand years ago, particularly mentions cradles in the last act and first scene of his *Amphitryon*; yet, Roman authors in general, have not written much respecting cradles, although minute in their descriptions of various couches, such as the *sella*, *lectica*, or *cathedra*. Dr. Kennedy inveighs bitterly against the custom of violently rocking children in cradles, and he shall be allowed to plead a cause, in which he seems so laudably interested.

"The agitations which infants sustain by the rocking of their cradles are regarded by many matrons and other equally sage observers as having very beneficial effects on their health and comfort. All agitations of this kind, however, must be gentle indeed if they pass not the point at which they are simply harmless. It is no argument in favour of rocking it in a cradle, that a crying child becomes silent immediately on this charming operation being resumed or continued:—the fact only proves that infants are quite susceptible of being trained to bad habits; and, like some of their mothers, can express with a smile their satisfaction when the desires arising out of such habits are gratified. If then, the mildest possible motion in a cradle be at best but harmless, it is not so with those violent agitations which multitudes of idle and unthinking nurses employ habitually with the design of lulling into quietness and silence, children crying aloud perhaps by reason of internal illness or of the torture inflicted by the inadvertent, though not the less cruel, misposition of a pin. Than such a practice, scarcely any thing can be more pernicious: it is a practice utterly inconsistant with humanity and, of course, in the highest degree reprehensible. The stillness that follows it, is seldom a manifestation of true and refreshing sleep:—it is oftener a kind of lethargic oppression induced by an irregular distribution of the blood in the brain. Violent rocking is particularly mischievous during the progress of teething:—its readiest effect then is, to produce convulsive affections which lead to fits of excessive drowsiness, to indigestion and emaciation; and, too often, in spite of the best treatment, terminate in death. Frequent repetition of it, moreover, seldom fails of determining blood in undue proportion to the brain, together with every sort of internal disorder; and, in an especial manner, of originating accumulations of water in the head, with all their most excruciating and deadly accompaniments. Were it, therefore, for no other reason than the prevention of these inconsiderate and fatal doings, the use of cradles should be altogether abolished. As an inducement to their admitting this necessity, it might be well if all mothers, who are partial to the practice of briskly rocking their children for the sake of silencing them, would but for one half-hour only submit to become themselves the objects of such delightful discipline;—and if, after this, they continue to be enamoured of the exercise, it will of course be natural for them to desire imparting the benefits of it to the darlings of their affection." 150.

It is very true that adults, when rocked in a cradle, successively experience sickness, ringing in the ears, giddiness, headache, and vomiting. Instances are known, wherein this act, the consequene of idle levity, induced a state of lethargy, which terminated in brain fever, and long resisted appropriate and vigorous treatment. Now, if mothers recollect, urges our intelligent author, that the brain, in grown individuals, is much firmer, and less susceptible of mechanical impression, than in infants and children, they will be convinced, that what produces such injuries on a mature organ, must inflict incalculable mischief on one which is tender and incomplete. He affirms, and without any qualification, that multitudes of children, especially in the inferior classes of society, perish, at a premature age, by diseases, having their first origin in the rough rockings employed by nurses, with the view of promoting an unnatural inclination to sleep. If this be really the case, and it is impossible to question such respectable authority, the practice should for ever be abolished, and cradles either altogether proscribed in families, or only used simply as small and convenient beds. We have no experience on this subject, and decline the expression of any opinion; but it is our duty to invite public attention to it, which it is hoped, will decide in the cause of truth and humanity. Our author may be prejudiced against the rocking of cradles, just as Mr. Else, of St. Thomas's Hospital, denied the uterus to be muscular,* yet asserted it to be so, when a small (and by him unsuspected) piece of it was designedly presented for his in-

---

* The best anatomists admit the muscularity of the uterus. Malpighi, Morgagni, Mery, Littre, Astruc, Ruysch, Monro, Vieusseus, Haller, and Haighton entertained a similar opinion. Mr. Charles Bell has also written an excellent paper on this subject, in the 4th volume of the Medico-Chirurgical Transactions.—*Rev.*

spection; but judging of him, from his correct and philosophical mode of thinking and writing, we cannot think he is, and, therefore, attach great weight to his sentiments.

Dr. Kennedy enters largely and elaborately into the qualities of the different milk of animals, the effects of air and exercise of every kind, comprehending walking, running, dancing, and certain playful games: also clothing, the textures and preparations of it, in a word, a multiplicity of interesting subjects, and for the whole of which the reader is unavoidably referred to the work. We had intended to offer a specimen or two, illustrative of the author's scientific manner, but we refrain, from a conviction that it is superfluous, inasmuch as, from the very low price of the book, and its small type and closely-filled pages, which contain a vast quantity of matter, it will be much sought after, and extensively read. It may be asked, and even justly asked, presuming that we have done this writer only common justice, where was the necessity for such a superior and scientific work, when it is avowedly addressed to mothers and nurses? Dr. Kennedy can best answer this question; we have already said, the title-page does him injustice; our opinion is, that he absolutely wrote the major part for the guidance and instruction of youthful practitioners, but that the natural modesty of the man, led him to mask his design, lest he should disappoint expectation, since it is undeniable, that many, very many, parts of his work are far above the comprehension of women. There are, however, other parts, expressly written for them, which they may read and study with pleasure and advantage; and, by observing the rules inculcated, mothers may hope to see their children improve in health and vigour, and, if females, in personal beauty, which is so anxiously wished for, and ardently admired by all ranks of society.

> "The teeming mother, anxious for her race,
> Begs for each birth the fortune of a face;
> Yet Vane could tell what ills from beauty spring:
> And Sedley cursed the form that pleased a king."
>
> *Johnson's Satire on the Vanity of Human Wishes.*

We hope we have written enough to obtain for Dr. Kennedy's production an attentive and dispassionate perusal; we are persuaded it needs nothing more to insure a favourable reception from his professional brethren, who, as a body, are ever candid and liberal to well meant endeavours, and successful exertions. To mothers and nurses, we venture to offer no advice; they cannot but patronize, and even admire, a book which gallantly inscribes them on its title-page;—to the junior branches of the profession, especially those living in the country, and engaged in the practice of midwifery, we, presuming on our years, strongly recommend it as a most valuable guide, not, indeed, in the higher walks of practice, but in the scarcely less important matters, which appertain to puerperal women, and their offspring. It only remains that we should take leave of Dr. Kennedy, which we do, with kindness and respect:—to us, he is a perfect stranger;* bias we can have none; truth is our only object—and, under its influence, we pronounce him to be a correct writer, an acute physiologist, and an excellent practitioner.

* The reviewer lives in a remote provincial city. To the principal Conductors of this Journal, Dr. K. is well known, as indeed to the Profession generally, by his writings.

